# A comparative study on anti-MOG and anti-AQP4 associated optic neuritis following mild COVID-19: insights from a Chinese single-center experience

**DOI:** 10.3389/fneur.2024.1416493

**Published:** 2024-06-26

**Authors:** Liang Sun, Jiawei Wang, Qinglin Yang, Yanjun Guo

**Affiliations:** Department of Neurology, Beijing Tongren Hospital, Capital Medical University, Beijing, China

**Keywords:** optic neuritis, severe acute respiratory syndrome coronavirus 2, coronavirus disease 2019, aquaporin-4 antibody, myelin oligodendrocyte glycoprotein antibody

## Abstract

**Background:**

Research on the relationship between mild COVID-19 and the subsequent development of isolated optic neuritis (ON) with antibodies specific to myelin oligodendrocyte glycoprotein (MOG-ON) and aquaporin 4 (AQP4-ON) is limited, particularly case–control studies that directly compare these conditions within the same affected population.

**Methods:**

A retrospective analysis of initial MOG-ON and AQP4-ON cases during the COVID-19 peak and subsequent months. Patients were classified as possible COVID-19 related ON (PCRON) or non-COVID-19 related ON (NCRON). The study compared epidemiology, comorbidities, and clinical features between these groups.

**Results:**

Patients with MOG-ON tended to develop ON symptoms closer in time to a mild COVID-19 infection compared to those with AQP4-ON (6.87 ± 6.25 weeks vs. 11.06 ± 5.84 weeks; *p* = 0.038), a significantly higher proportion of patients with MON-ON developing symptoms within 6 weeks after COVID-19 compared to those with AQP4-ON (15/23 [65.2%] vs. 5/17 [29.4%]; *p* = 0.025). Comparing MOG-ON and AQP4-ON patients, MOG-ON patients were more likely to have a recent infection before ON onset (73.1% vs. 30%; *p* = 0.007) and had better peak and post-treatment visual acuity (*p* = 0.01; *p* < 0.001). In contrast, AQP4-ON patients frequently showed comorbid connective tissue diseases (30.0% vs. 0%, *p* = 0.004) and antinuclear antibody abnormalities (40.0% vs. 7.7%, *p* = 0.012). Among MOG-ON patients, PCRON had increased rates of atherosclerotic vascular diseases (AVDs) (53.3% vs. 9.1%, *p* = 0.036), phospholipid antibody abnormalities (60.0% vs. 18.2%, *p* = 0.04), and bilateral visual impairment (66.7% vs. 9.1%, *p* = 0.005). Multivariate analysis pinpointed AVDs (OR = 15.21, *p* = 0.043) and bilateral involvement (OR = 25.15, *p* = 0.015) as independent factors related to COVID-19 associated MOG-ON, with both being good discriminators for PCRON (AUC = 0.879). No differences were found between the PCRON and NCRON groups in AQP4-ON patients.

**Conclusion:**

Mild COVID-19 is more likely associated with MOG-ON than AQP4-ON. MOG-ON that develops within 6 weeks following a COVID-19 infection may be associated with the COVID-19 infection. AVDs may have a synergistic effect on MOG-ON in patients with COVID-19, which warrants further investigation. COVID-19 related MOG-ON often affects both eyes, and acute visual function damage can be severe, but generally has a good prognosis.

## 1 Introduction

A significant Coronavirus Disease 2019 (COVID-19) epidemic has emerged in China following the termination of the dynamic zero COVID-19 strategy, with 82.4% of the Chinese population being infected from December 2022 to February 7, 2023 (1), and neurological manifestations related COVID-19 are being reported increasingly (2). However, identifying neurological diseases associated with COVID-19 in patients with mild or asymptomatic respiratory infections can be challenging. This is particularly true if the primary COVID-19 illness occurred weeks prior, likely due to limited awareness of such complications and the significant delay between infection and the onset of neurological symptoms.

Dozens of cases of Optic Neuritis (ON) have been reported in patients following infection with Severe Acute Respiratory Syndrome Coronavirus 2 (SARS-CoV-2) since the onset of the COVID-19 pandemic in December 2019 (3–5). Additionally, cases of Aquaporin-4 Antibody (AQP4-Ab) positive Neuromyelitis Optica Spectrum Disorder (NMOSD) and Myelin Oligodendrocyte Glycoprotein Antibody (MOG-Ab)-associated Disease (MOGAD) have also emerged post-COVID-19 vaccination or infection (6–13), suggesting that SARS-CoV-2 infection may precipitate the onset of ON or even the production of MOG-Ab and AQP4-Ab (14). However, research exploring the connection between COVID-19 and ON linked to antibodies against MOG Immunoglobulin G (IgG) (MOG-ON) and AQP4 IgG (AQP4-ON) is limited. The current literature primarily comprises isolated case reports and comprehensive literature reviews, with a notable absence of case–control studies conducted within the same affected populations. This makes it difficult to determine the exact association between COVID-19 and both MOG-ON and AQP4-ON, as well as their actual prevalence and distinctive characteristics.

Here, we investigate patients who developed their initial symptoms of AQP4-ON and MOG-ON during and after the COVID-19 pandemic in China. We perform a comparative analysis to examine the influence of COVID-19 on the development of MOG-ON and AQP4-ON, as well as to identify distinctive features associated with COVID-19-related ON.

## 2 Methods

This study is a single-center case retrospective study, with the research subjects being the initial, isolated attack MOG-ON and AQP4-ON patients who were hospitalized and treated in the neurology department of Beijing Tongren Hospital, Capital Medical University from December 15, 2022 to June 30, 2023. The diagnosis was made by two or more neuro-ophthalmology experts. The diagnosis and classification of optic neuritis (ON) were based on the diagnostic and classification criteria for ON developed by the 2022 international expert group (15). ① The diagnosis of optic neuritis meets the definite diagnostic criteria for optic neuritis; ② MOG-ON diagnosis requirements: (a) Laboratory examination: serum MOG-IgG positive based on cell-based indirect immune-fluorescence assay (CBA); (b) meets the diagnostic criteria for optic neuritis; and (c) Exclude other diagnoses. ③ AQP4-ON: The requirements are consistent with the diagnosis of optic neuritis, and serum AQP4-IgG is detected as positive using CBA method.

To determine a history of COVID-19, the following criteria were used: Individuals who presented with symptoms such as fever, cough, or sore throat, and tested positive for SARS-CoV-2 through either a reverse transcription-polymerase chain reaction (RT-PCR) or antigen tests using nasopharyngeal or throat swabs, were classified as having a history of COVID-19.

According to the framework published in Lancet Neurol for investigating patients with suspected COVID-19-associated neurological disease (2), ON patients who develop symptoms within 6 weeks after SARS-CoV-2 infection were considered as Possbile-COVID-19 Related ON (PCRON). Patients with MOG-ON and AQP4-ON, who developed these conditions more than 6 weeks post-COVID-19 infection, as well as those lacking a pre-COVID-19 history, have been categorized as having Non-COVID-19 Related ON (NCRON). Exclusion criteria were not meeting the diagnostic criteria for MOG-ON or AQP4-ON, having recurrent MOG-ON or AQP4-ON, or having an uncertain time relationship between SARS-CoV-2 infection and the onset of MOG-ON or AQP4-ON.

For each included patient, Potential inciting events preceding development of neurological symptoms were recorded including infections, past medical history and the pregnancy and delivery status at the time of disease onset. We also analyzed the open-ended answers provided by patients regarding possible inducing factors for self-evaluation. Atherosclerotic vascular diseases (AVD)and their risk factors were recorded, including hypertension, diabetes, atherosclerotic vascular stenosis, coronary heart disease, and cerebrovascular disease. The patient’s history of connective tissue diseases (CTDs) and related antibodies was also analyzed, including the results of rheumatism triad tests, thyroid-related antibodies, ESR, antinuclear antibody panel, and antiphospholipid antibody spectrum, all conducted during hospitalization.

As for clinical characteristics, the visual examination involved an appearance refraction test to detect the patient’s best corrected visual acuity and utilized the international standard visual acuity chart to obtain decimal scoring method visual acuity. When a patient’s monocular vision deteriorated, we analyzed the peak visual acuity (worst visual acuity) of the affected eye and the visual acuity after treatment. Similarly, when a patient’s binocular vision declined, we assessed the peak visual acuity of the eye with the most severe visual impairment and the visual acuity after treatment. Visual acuity is classified into six grades: ≥0.8, <0.8–0.5, <0.5–0.3, <0.3–0.1, <0.1–0.05, and < 0.05, according to the 2019 World Health Organization (WHO) visual impairment criteria (16). MRI images were reviewed by an independent reviewer blinded to the final diagnosis for detailed analysis. Patients underwent optic nerve MRI using a 3.0 T scanner. The MRI protocols included 3-mm thick T1 and T2-weighted sequences with fat suppression, before and after gadolinium administration. We identified higher signals within the optic nerve on T2-weighted fat suppression images and neural and/or perineural enhanced lesions on T1-weighted fat suppression images. Abnormal signals and enhancement were observed in the optic disc, intraorbital, intracanalicular, and intracranial segments of the optic nerve on MRI. The proportion of optic nerve sheaths enhancement was also recorded. Simultaneously, the presence or absence of MRI lesions was evaluated. Cerebrospinal fluid tests were also conducted, and information regarding the patient’s treatment was collected.

All data were processed through IBM SPSS Statistics 25.0 for Windows (SPSS Inc., Chicago, IL, United States). Normally distributed quantitative data were expressed as mean ± standard deviation. Statistical analysis of quantitative data was performed using independent sample *T* test for two groups. The count data were expressed as number (%) and analyzed by chi-square test. Fisher’s exact test was used when the expected cell count was <5. The rank data were analyzed by rank sum test. *p* ≤ 0.05 indicated that the difference was statistically significant. Multivariate analysis of binary classification was performed by Logistic regression analysis. Graphs were generated using GraphPad Prism 9.5.0.

## 3 Results

**Figure 1:**
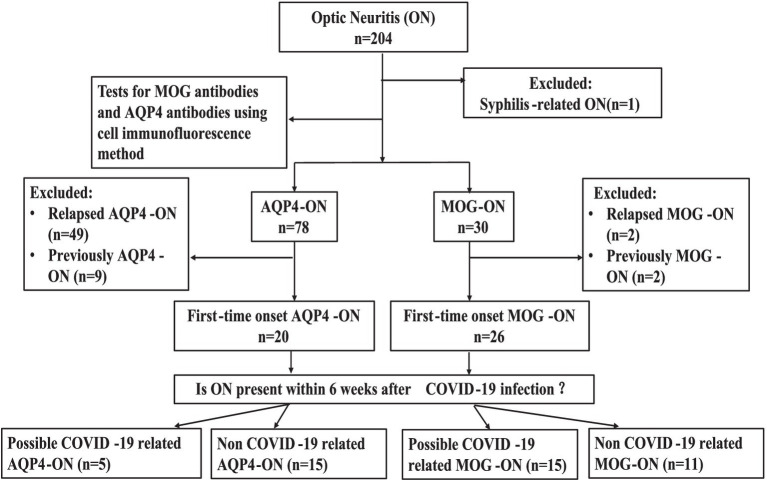
Participant screening and group assignment. *n*, number; ON, optic neuritis; MOG-ON, antibodies against myelin oligodendrocyte glycoprotein immunoglobulin G (IgG) associated ON; AQP4-ON, antibodies against aquaporin 4 IgG associated ON.

A total of 204 patients were identified using “ON” as the primary diagnosis. Except for one patient who was diagnosed with syphilis-related ON, all patients underwent tests for MOG antibodies and AQP4 antibodies using cell immunofluorescence method. 30 patients had positive MOG antibodies and 78 patients had positive AQP4 antibodies.2 patients with relapsed MOG-ON, 49 patients with relapsed AQP4-ON, and 11 patients with a previous medical history but no recurrence during this hospitalization (2 patients with MOG-ON,9 patients with AQP4-ON) were excluded. In summary, 26 patients with MOG-ON and 20 patients with AQP4- ON were enrolled. Details regarding the patient screening and grouping process are shown in Figure 1.

### 3.1 COVID-19 history

Among the 46 patients who initially presented with MOG-ON/AQP4-ON, 40 (87%) had a history of mild SARS-CoV-2 infection prior to the onset of their neurological symptoms. Neither hospitalization for COVID-19 nor treatments such as oxygen therapy, ventilator support, steroids, antivirals, or anticoagulants were required for these patients. All had recovered from the infection by the time they were hospitalized for MOG-ON/AQP4-ON. It’s noteworthy that all 40 patients contracted COVID-19 for the first time between December 1, 2022, and February 26, 2022. In addition, two MOG-ON patients underwent a second SARS-CoV-2 infection within 6 weeks before the onset of their disease, specifically on March 1, 2023, and May 6, 2023.

Analysis of the time relationship between COVID-19 and ON onset revealed that ON occurred between 1 to 20 weeks after COVID-19. The mean time from COVID-19 to ON onset was shorter in patients with MOG-ON compared to patients with AQP4-ON (6.87 ± 6.25 weeks vs. 11.06 ± 5.84 weeks; *p* = 0.038), indicating a closer temporal association between MOG-ON onset and COVID-19. Using a contingency table chi-square test, the results showed that there was a significantly higher proportion of patients with MON-ON developing symptoms within 6 weeks after COVID-19 compared to those with AQP4-ON (15/23 [65.2%] vs. 5/17 [29.4%]; *p* = 0.025). Details and vaccination status of COVID-19 were showed in Table 1.

**Table 1:** COVID-19 infection and vaccination status.

	MOG-ON (*n* = 26)	AQP4-ON (*n* = 20)	*p-*value
COVID-19 history positive (*n*, %)	23 (88.5%)	17 (85.0%)	0.730
Interval from COVID-19 infection to ON [Weeks (mean ± SD) (range)]	6.87 ± 6.25 (1 ~ 20)	11.06 ± 5.84 (1 ~ 20)	0.038
Developed within 6 weeks post-COVID-19 infection (*n*, %)	15 (65.2%)	5 (29.4%)	0.025
Vaccination status of COVID-19 (vaccinated: unvaccinated: unknown)	21:1:4	14:3:3	0.408

*n*, number; ON, optic neuritis; MOG-ON, antibodies against myelin oligodendrocyte glycoprotein immunoglobulin G (IgG) associated ON; AQP4-ON, antibodies against aquaporin 4 IgG associated ON.

### 3.2 Epidemiologic data

There was no statistically significant difference in age, gender, pregnancy, and delivery status between MOG-ON and AQP4-ON patients. The male-to-female ratio was 11:15 for MOG-ON patients and 5:15 for AQP4-ON patients. Among the MOG-ON patients, 3 out of 26 developed the disease during the second trimester or in the postpartum period. Additionally, 1 out of 20 AQP4-ON patients exhibited symptoms just 10 days after childbirth (Table 2).

**Table 2:** Demographics, comorbidities, and clinical features in AQP4-ON vs. MOG-ON patients.

	MOG-ON (*n* = 26)	AQP4-ON (*n* = 20)	*p-*value
Age (mean ± SD),y	44.12 ± 16.11	49.30 ± 15.19	0.274
Gender (M:F)	11:15	5:15	0.222
AVD and risk factors (*n*, %)	9 (34.6%)	6 (30.0%)	0.741
Comorbid CTDs (*n*, %)	0	6 (30.0%)	0.004
Positive antinuclear antibodies (*n*, %)	2 (7.7%)	8 (40.0%)	0.012
Positive phospholipid antibodies (*n*, %)	11 (42.3%)	5 (25.0%)	0.222
Positive thyroid-related antibodies (*n*, %)	3 (11.5%)	7 (35.0%)	0.077
Preceding infections within 6 weeks (*n*, %)	19 (73.1%)	6 (30.0%)	0.004
During pregnancy or postpartum (*n*, %)	3 (11.5%)	1 (5.0%)	0.622
Ocular pain (*n*, %)	22 (84.6%)	17 (85.0%)	1.000
Bilateral visual impairment (*n*, %)	11 (42.3%)	5 (25.0%)	0.222
Peripapillary hemorrhage (*n*, %)	7 (26.9%)	1 (5.0%)	0.113
Peak visual acuity M(P25, P75)	6 (4.0,6.0)	6 (6.0,6.0)	0.001
Post-treatment visual acuity M(P25, P75)	1 (1.0,3.3)	6 (4.3,6.0)	0.001
IVMP treatment (*n*, %)	26 (100.0%)	18 (90.0%)	0.184
Immunosuppressants/IVIG (*n*, %)	9 (34.6%)	12 (60%)	0.087
≥2 sites optic nerve lesions on MRI (*n*, %)	15 (57.7%)	10 (50.0%)	0.822
Optic sheath affection on MRI (*n*, %)	7 (26.9%)	3 (15.0%)	0.476

*n*, number; ON, optic neuritis; MOG-ON, antibodies against myelin oligodendrocyte glycoprotein immunoglobulin G (IgG) associated ON; AQP4-ON, antibodies against aquaporin 4 IgG associated ON; AVD, atherosclerotic vascular diseases; CTDs, connective tissue diseases; IVMP, intravenous methylprednisolone; IVIG, intravenous immunoglobulin; M, median; P25, first quartile; P75, third quartile.

In comparing PCRON and NCRON, it’s noteworthy that the gender distribution was roughly equivalent among patients with PCRON, exhibiting an 8:7 male-to-female ratio for MOG-ON patients and a 3:2 male-to-female ratio for AQP4-ON patients. In contrast, female patients with NCRON appeared to exhibit a higher incidence in both cohorts, with a 3:8 male-to-female ratio for MOG-ON patients and a 2:13 male-to-female ratio for AQP4-ON patients. However, these differences did not reach statistical significance. Among the four patients who experienced disease onset during pregnancy and postpartum, one case from the MOG-ON cohort and one case from the AQP4-ON cohort were classified as PCRON.

### 3.3 Concomitant diseases

Upon comparing MOG-ON and AQP4-ON patients, significant differences were observed regarding connective tissue diseases (CTDs) and related antibodies. A higher percentage of AQP4-ON patients had comorbidities such as Sjögren’s syndrome and systemic lupus erythematosus compared to MOG-ON patients (6/20 [30.0%] vs. 0/26[0.0%]; *p* = 0.004). The AQP4-ON cohort also exhibited a notably greater prevalence of an abnormal antinuclear antibody spectrum (8/20 [40.0%] vs. 2/26[7.7%]; *p* = 0.012), characterized by increased levels of antinuclear antibody, anti-SSA, anti-SSB, anti-RO-52, anti-centromere antibody, and antimitochondrial antibody. However, there was no significant disparity between the two groups in terms of abnormal thyroid-related antibodies, even though a subset of patients from both groups showed positivity for these antibodies. Besides, the incidence of comorbid AVD and associated risk factors did not differ statistically between MOG-ON and AQP4-ON patients. In terms of history of preceding infection, a higher proportion of MOG-ON patients reported a history of COVID-19 or other infections within 6 weeks prior to the onset of ON compared to the AQP4-ON group (19/26 [73.1%] vs. 6 /20 [30%]; *p* = 0.007). Additionally, three MOG-ON patients experienced fatigue preceding their illness, and one patient from the AQP4-ON group was diagnosed with lung cancer a year before developing neurological symptoms (Table 2).

In a comparison between the PCRON and NCRON groups among patients with MOG-ON, Fisher’s exact test indicated that there was a significantly higher proportion of comorbid AVD and associated risk factors within the PCRON group (8/15 [53.3%] vs. 1/11[9.1%]; *p* = 0.036). The most common underlying conditions identified were hypertension (9 cases), diabetes (6 cases), cerebrovascular disease (4 cases), coronary heart disease, and other AVDs (5 cases). Remarkably, those in the PCRON group demonstrated a substantially increased prevalence of abnormalities in the phospholipid antibody spectrum compared to their NCRON counterparts (9/15 [60.0%] vs.2/11 [18.2%]; *p* = 0.04), indicating elevated β2 glycoprotein I antibodies, reduced protein S levels, lowered antithrombin III activity, and heightened LA1/LA2 levels. In contrast, among patients with AQP4-ON, there were no notable disparities in rheumatic immune markers or the presence of comorbid AVDs and associated risk factors when comparing the PCRON and NCRON groups (Table 3).

**Table 3:** Comparison of patients with PCRON and NCRON: epidemiologic profiles and associated comorbidities.

	MOG-ON			AQP4-ON		
	PCRON (15)	NCRON (11)	*p-*value	PCRON (5)	NCRON (15)	*p-*value
Age (mean ± SD), y	26 ~ 69 (49.27 ± 14.75)	15 ~ 63 (37.09 ± 15.83)	0.055	26 ~ 70 (43.00 ± 19.21)	30 ~ 76 (51.40 ± 13.74)	0.296
Gender (M:F)	8:7	3:8	0.246	3:2	2:13	0.073
AVD and risk factors (*n*, %)	8 (53.3%)	1 (9.1%)	0.036	1 (20%)	5 (33.3%)	1.000
Comorbid CTDs (*n*, %)	0	0	–	1 (20%)	5 (33.3%)	1.000
Positive thyroid-related antibodies (*n*, %)	2 (13.3%)	1 (9.1%)	1.000	1 (20%)	6 (40%)	0.613
Positive phospholipid antibodies (*n*, %)	9 (60.0%)	2 (18.2%)	0.040	1 (20%)	4 (26.7%)	1.000
Positive antinuclear antibodies (*n*, %)	1 (6.7%)	1 (9.1%)	1.000	0	8 (53.3%)	0.055
Preceding infections within 6 weeks (*n*, %)	15 (100%)	4 (36.4%)	0.001	5 (100%)	1 (6.7%)	0.001
During pregnancy or postpartum (*n*, %)	1 (6.67%)	2 (18.2%)	0.556	1 (20%)	0	0.250

PCRON, possible-COVID-19 related ON; NCRON, non-COVID-19 related ON; *n*, number; ON, Optic Neuritis; MOG-ON, antibodies against myelin oligodendrocyte glycoprotein immunoglobulin G (IgG) associated ON; AQP4-ON, antibodies against aquaporin 4 IgG associated ON; AVD, atherosclerotic vascular diseases; CTDs, connective tissue diseases.

### 3.4 Clinical presentation

**Figure 2:**
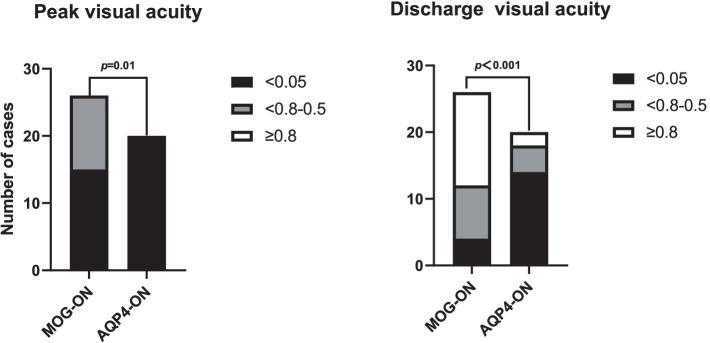
Comparison of the peak visual acuity and discharge visual acuity between MOG-ON and AQP4-ON patients. ON, optic neuritis; MOG-ON, antibodies against myelin oligodendrocyte glycoprotein immunoglobulin G (IgG) associated ON; AQP4-ON, antibodies against aquaporin 4 IgG associated ON.

**Figure 3:**
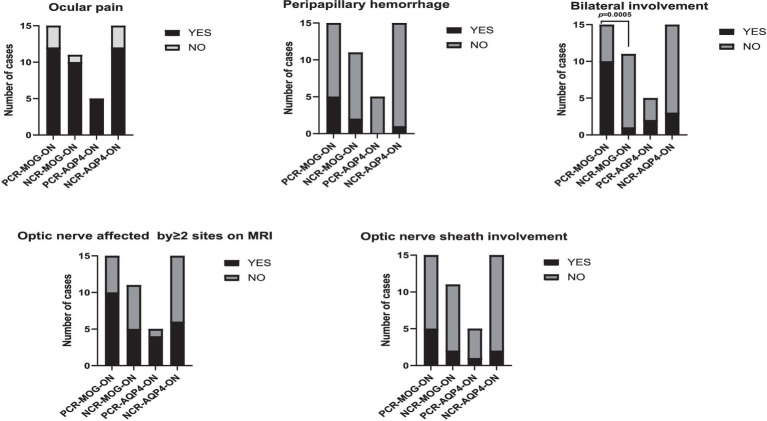
Comparing PCRON and NCRON among MOG-ON and AQP4-ON patients: clinical profiles. PCR-MOG-ON, possible COVID-19 related MOG-ON; NCR-MOG-ON, non-COVID-19 related MOG-ON; PCR-AQP4-ON, possible COVID-19 related AQP4-ON; NCR-AQP4-ON, non-COVID-19 related AQP4-ON.

As for the clinical characteristics of MOG-ON and AQP4-ON patients, no significant differences were found in the incidence of bilateral visual impairment (11/26[42.3%] vs. 5/20[25%]; *p* = 0.222) or eye pain (22/26[84.6%] vs. 17/20[85.0%]; *p* = 1.000). The rank sum test was utilized for comparison of visual acuity, and the results indicated that MOG-ON patients had superior peak visual acuity (Z = -3.262, *p* = 0.01) and post-treatment visual acuity compared to AQP4-ON patients(Z = -4.036,*p* < 0.001). Specifically, Both MOG-ON and AQP4-ON patients exhibited a high prevalence of severe peak visual acuity impairment, with 57.7% of MOG-ON patients (15/26) and all AQP4-ON patients (20/20) having a peak visual acuity less than 0.05. However, after treatment, 14/26 (53.8%) MOG-ON patients achieved visual acuity of 0.8 or better, and only 4/26 cases (15.4%) had discharge visual acuity remaining below 0.05 (Figure 2). Perivascular hemorrhage around the optic disc seems more common in MOG-ON patients but with no statistical significance (26.9% vs. 5% in AQP4-ON; *p* = 0.113). On MRI, over half of both groups exhibited optic nerve involvement in≥2 areas, with a non-significant difference in optic sheath affection rates (7/26[26.9%] vs. 3/20[15.0%]; *p* = 0.476). In the MOG-ON cohort, the PCRON group exhibited a significantly elevated rate of bilateral visual impairment compared to the NCRON group (10/15 [66.7%] vs. 1/10 [9.1%]; *p* = 0.005). Conversely, in the AQP4-ON patients, no significant disparity emerged between the PCRON and NCRON groups (2/5 [40.0%] vs. 3/15 [20.0%]; *p* = 0.560). Additionally, no statistical differences were observed in peak visual acuity and post-treatment visual acuity between PCRON and NCRON in both the MOG-ON cohort (Z = −0.406, *p* = 0.721; Z = −1.162, *p* = 0.305) and AQP4-ON cohort (Z = 0.001, *p* = 1.000; Z = −0.485, *p* = 0.628). MRI analyses indicated no significant distinctions in the extent of optic nerve involvement or the ratio of patients with optic nerve sheaths affected between the PCRON and NCRON groups, neither in MOG-ON patients (5/15[33.3%] vs. 2/11[18.2%]; *p* = 0.658) nor in AQP4-ON patients (1/5[20.0%] vs. 2/15[13.3%]; *p* = 1.000). Although there was no significant difference in the occurrence of peripapillary hemorrhage across the groups. In the MOG-ON cohort, 5 out of 7 patients with peripapillary hemorrhage were in the PCRON group (Figure 3).

29 patients underwent cerebrospinal fluid (CSF) examination, including 19 patients with MOG-ON and 10 patients with AQP4-ON. In the MOG-ON group, one PCRON patient had an elevated cerebrospinal fluid white cell count of 30 *10^6^/L, and a different RCRON patient showed an increased cerebrospinal fluid protein level of 89.8 mg/dL, while the other patients exhibited normal levels of both. Additionally, one NCRON patient tested positive for oligoclonal bands (OCB), but all patients with MOG-ON had a normal 24 h IgG synthesis rate. In the AQP4-ON group, the cerebrospinal fluid white cell count and protein level were within normal limits. All patients tested negative for OCB. Two patients had mildly elevated 24 h IgG synthesis rates (1 PCRON patient at 4.86 mg/Day and 1 NCRON patient at 5.96 mg/Day). A total of 3 patients underwent cerebrospinal fluid COVID-19 nucleic acid testing, and the results were negative.

### 3.5 Treatment during hospitalization

All patients with MOG-ON (26/26) and 90% of patients with AQP4-ON (18/20) were treated with intravenous methylprednisolone (IVMP) 500 mg or 1,000 mg per day for 3 or 5 days based on the severity of vision loss and followed by tapered oral prednisone therapy. The 2 AQP4-ON patients who did not receive IVMP treatment belong to the NCRON group.

In all, 34.6% (9/26) of MOG-ON patients and 60% (12/20) of AQP4-ON patients received further immunosuppressants or intravenous immunoglobulin (IVIG) treatment during hospitalization. For patients with MOG-ON, 8 received Mycophenolate mofetil (MMF) and one patient also received IVIG, Another patient received Rituximab. For patients with AQP4-ON, 10 received MMF, one received Inebilizumab, and another received IVIG (Table 2).

### 3.6 Independent related factors for PCRON

**Figure 4:**
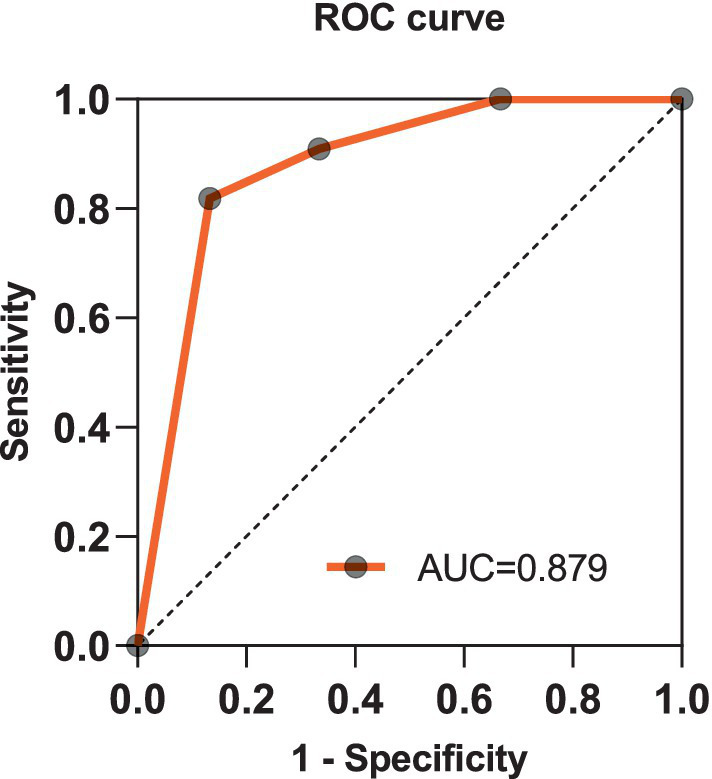
Discriminating PCRON in MOG-ON patients using ROC curve metrics.

We used PCRON and NCRON as the binary dependent variable in the Logistic Regression Analysis of patients with MOG-ON. The independent variables included comorbid AVDs, abnormalities in the phospholipid antibody spectrum, and bilateral visual impairment, which have been filtered out through the previous single-factor analysis and show statistically significant differences. In the multivariate analysis, the combined abnormalities in the phospholipid antibody spectrum are no longer an independently related factor (*p* = 0.591). However, the presence of AVDs (Odds Ratio [OR] = 15.21, 95%CI 1.09–213.14, *p* = 0.043) and bilateral involvement (OR = 25.15, 95%CI 1.94–326.38, *p* = 0.014) remain as significant independent correlates for PCRON in MOG-ON patients. The Receiver Operating Characteristic (ROC) curves reveal that comorbid AVDs and bilateral involvement are good discriminating factors for PCRON in MOG-ON patients (Area Under Curve[AUC] = 0.879). as shown in Figure 4.

Univariate analysis failed to detect differences between PCRON and NCRON among patients with AQP4-ON; consequently, we opted not to conduct Logistic Regression Analysis on this patient group.

## 4 Discussion

Genetic predisposition and environmental factors are identified as the primary risk contributors to demyelinating disorders. Parainfectious pathogenesis as an environmental risk factor could play a key role in triggering conditions such as NMOSD (17, 18) and MOGAD (19, 20). With the widespread outbreak of COVID-19, studies have reported cases of isolated ON following COVID-19 infection in patients who were seropositive for MOG-Ab and AQP4-Ab. However, case–control studies examining the link between mild COVID-19 and isolated optic neuritis (ON) associated with MOG-Ab andAQP4-Ab within the same population are scarce. This scarcity results in limitations in multivariate analyses aimed at ruling out confounding factors, establishing a causal link between COVID-19 and MOG-ON/AQP4-ON, and understanding the underlying pathogenesis and prognosis.

The termination of the dynamic zero COVID-19 strategy and the subsequent outbreak epidemic of COVID-19 in China has provided us a chance to investigate the relationship between COVID-19 and ON. To eliminate confounding factors such as steroid and immunosuppressant treatment, this study focused on MOGAD and NMOSD patients with initial onset and clinical manifestations of isolated ON. Although isolated ON is a relatively localized change in central nervous system demyelination, it has clear and easily quantifiable clinical symptoms. In contrast, patients with COVID-19 may have presented with mild symptoms a few weeks ago and could have already recovered by the time of admission, this complexity makes studying the relationship between COVID-19 and ON somewhat challenging. This study found a significant difference in the temporal correlation between SARS-CoV-2 infection and MOG-ON and AQP4-ON during the pandemic. Although the time interval between COVID-19 infection and the subsequent onset of ON has varied among reported cases, based on the experience of neurological associations with COVID-19 (2), we have categorized ON occurring within 6 weeks after SARS-CoV-2 infection as likely related to COVID-19. This timeframe seems to effectively differentiate AQP4-ON from MOG-ON, suggesting that a six-week period could serve as an appropriate benchmark for temporal correlation. Compared to patients with AQP4-ON, those with MOG-ON experienced a shorter interval from COVID-19 infection to symptom onset. Specifically, a significantly higher proportion of MOG-ON patients exhibited symptoms within 6 weeks of the COVID-19 infection compared to AQP4-ON patients. This finding suggests a stronger association between COVID-19 infection and the onset of MOG-ON compared to AQP4-ON. This is corroborated by a recent prospective case series study (14) that identified 35 patients developing ON post-COVID-19 infection, and CBA revealed seropositive MOG-Ab in 10 cases and AQP4-Ab in 2 cases, respectively, of which 2 AQP4-Ab-seropositive cases and 1 MOG-Ab-seropositive case had a past medical history of ON. These results suggest a potential link between SARS-CoV-2 infection and the production of MOG antibodies. In Ontario, Canada, a study (21) found that population-wide infection control measures to halt SARS-CoV-2 spread in March 2020 significantly reduced MOGAD cases among children, while MS and AQP4-NMOSD rates remained unchanged. This indirectly supports a link between SARS-CoV-2 and the development of MOGAD. Analyzing SARS-CoV-2 serology, a higher prevalence of IgG antibodies was found in MOGAD patients compared to controls, providing preliminary evidence for SARS-CoV-2 as a potential trigger for MOGAD, despite the non-significant difference (22).

The pathophysiological mechanism underlying MOG-ON following COVID-19 infection remains unclear. In fact, Although the overall proportion of postinfectious cases is not known, MOGAD have been associated with a wide variety of infectious organisms, including influenza, Epstein–Barr virus, herpes simplex virus, SARS-CoV-2, human herpes virus, Borrelia, measles, Zika virus, *Mycoplasma pneumoniae* and unspecified infections. That the disease can arise in the aftermath of such diverse microorganisms argues against the sole mechanism being molecular mimicry and may instead suggest an epiphenomenon associated with robust immune activation and/or disruption of the blood–brain barrier (23). COVID-19 infection causes a dysregulated interferon response and increases the expression of several pro-inflammatory cytokines, including IL-1β, TNF and IL-6. This host response reached a critical threshold sufficient to activate MOG-IgG1 specific B-cells, leading to an increased titer of serum MOG antibodies. CD4 + and CD8+ T cell responses associated with the SARS-CoV-2 virus provide a stimulus for bystander activation and co-stimulation of autoreactive T- and B-cells (24). Infection and multiplication of SARS-CoV-2 in various organs lead to upregulate interferon signaling pathways in the neurovascular unit. This further causes dysregulation of the blood–brain barrier, resulting in the penetration of MOG antibodies into the CNS and leading to neuro-inflammatory disorders.

This study could not establish a correlation between COVID-19 and the initial onset of AQP4-ON due to weaker temporal association compared to MOG-ON, fewer patients developing the condition within 6 weeks post-infection, and the lack of distinctive features in the five cases that did develop the disease within this period. While A systematic review (25) identified 41 cases linking NMOSD to SARS-CoV-2 infection and COVID-19 vaccination, however, the symptom onset occurring between 3 and120 days post-infection, and transverse myelitis was the most common neurological manifestation. Furthermore, existing research appears to suggest a correlation between COVID-19 infection and NMOSD relapses. The Neuroimmunology Brazilian Study Group (26) compiled a report on 2,061 NMOSD patients monitored by neurologists participating in the registry, and identified SARS-CoV2 infection as a risk factor for NMOSD relapses. This assertion gained additional validation through a Mendelian randomization study, which disclosed that genetically forecasted SARS-CoV2 infection played a contributing role in NMO-IgG positive relapses (27).

This research identified certain characteristics among patients with MOG-ON who develop symptoms within 6 weeks after COVID-19 and provides additional evidence these patients may be associated with SARS-CoV-2 infection. Firstly, A high proportion of patients with atherosclerotic vascular disease and risk factors were observed, with approximately half of the COVID-19 patients who developed MOG-ON symptoms within 6 weeks having atherosclerotic vascular disease and its associated risk factors. Secondly, a high proportion of patients with abnormal antiphospholipid antibody spectrum was observed: 60% of the COVID-19 patients who developed MOG-ON symptoms within 6 weeks had abnormal antiphospholipid antibody spectrum, characterized by increased β2 glycoprotein antibody, decreased protein S, decreased antithrombin III percentage, and increased LA1/LA2 levels. Thirdly, the proportion of patients with bilateral eye involvement was high: 66.7% of the MOG-ON patients who developed symptoms within 6 weeks after COVID-19 had bilateral eye involvement, while only 9.1% of the other MOG-ON patients had this feature.

Existing research has revealed that COVID-19 infection can lead to abnormalities in the antiphospholipid antibody spectrum (28–30). In a study involving 29 severe COVID-19 patients, it was found that 68.7% of the patients with no history of autoimmune diseases tested positive for systemic autoantibodies, predominantly including antinuclear antibodies and anti β2 glycoprotein antibodies (34.5% each) (30). Chuan-bin Sun also detected Anti-β2-glycoprotein I IgM antibody and anticardiolipin IgM antibody in 3 and 2 cases, respectively, out of 35 cases of ON following SARS-CoV-2 infection, including one case that was MOG-Ab seropositive (14). Our findings suggest that patients with mild COVID-19 who developed MOG-ON also exhibited a high incidence of an abnormal antiphospholipid antibody spectrum, indicating that even a mild case of COVID-19 may trigger a positive antiphospholipid antibody response, which could potentially serve as a marker for subsequent ON or even MOG-ON. In the multifactorial analysis, vascular disease and its risk factors superseded abnormalities in antiphospholipid antibody spectrum as independent related factors for COVID-19-related MOG-ON. This suggests that the abnormality of the antiphospholipid antibody spectrum in patients with COVID-19-related MOG-ON may arise from underlying vascular endothelial damage. To the best of our knowledge, this study is the first to report that AVD and elevated abnormal antiphospholipid antibody spectrum may act synergistically in patients with COVID-19 to develop MOG-ON. These findings provide new insights into the pathogenesis of COVID-19 leading to MOGAD and warrant further investigation.

In examining the clinical features of patients with MOG-ON potentially linked to COVID-19, a key distinction from typical MOG-ON cases was noted: both genders were affected at a similar rate, and there was a greater likelihood of the condition affecting both eyes. Despite this, the peak and discharge visual acuities did not significantly differ from non-COVID-19 related MOG-ON cases. At their worst, patients suffered from severe visual impairment, with around 73% experiencing significant vision loss (<0.1). While all patients showed improvement in visual acuity upon discharge, a minority (13 to 18%) still endured considerable visual impairment. Pain presence, fundus appearance, and the extent of optic nerve involvement on MRI scans were comparable to those with NCRON. which is consistent with typical MOGAD presentation (20, 31–33). In the subset of MOG-ON patients with PCRON, some exhibited abnormal cerebrospinal fluid findings, including elevated protein and mononuclear cells. This suggests that PCRON may be more likely to present with cerebrospinal fluid abnormalities than NCRON. Further follow-up is needed to assess patients’ outcomes and recurrence rates, in order to determine whether COVID-19 associated MOG-ON has unique clinical features.

In our study, we also compared the characteristics of MOG-ON and AQP4-ON, corroborating earlier findings on their distinct traits. We found no significant differences in age, gender, or the prevalence of AVD and risk factors between the two patient groups. Notably, a higher percentage of MOG-ON patients had a history of preceding nonspecific infections, with 73.1% reporting such an occurrence before symptom onset. This aligns with Dubey et al.’s suggestion that MOGAD may be associated with non-specific viral infections (20). The University of Florida research also identified common triggers like flu-like illnesses and pregnancy more frequently in MOGAD than in NMOSD patients (34). In our study, three MOG-ON patients were either pregnant or postpartum, while one AQP4-ON patient was in the postpartum period and had a history of systemic lupus erythematosus. Whether COVID-19 and pregnancy/delivery interact to influence the development of MOG-ON and AQP4-ON requires further investigation with larger cohorts. Conversely, AQP4-ON patients more frequently had comorbidities such as Sjögren’s syndrome and systemic lupus erythematosus, along with abnormalities in antinuclear antibody spectrum immunological indicators. Our observations revealed that AQP4-ON patients experienced worse peak visual acuity and post-treatment visual outcomes compared to MOG-ON patients, consistent with prior findings (34). Additionally, a greater proportion of MOG-ON patients seemed to have peripapillary hemorrhage and optic nerve sheath involvement, although our sample size limited our ability to detect statistical significance. No differences were evident in the optic nerve MRI features between the two conditions. In CSF analysis, we observed that only 2 out of 19 MOG-ON patients presented with elevated leukocytes and protein, while all 10 AQP4-ON patients exhibited normal findings. This frequency of abnormalities appears lower than previously reported, especially for MOG-ON patients (35). The divergence might be attributed to the limited CNS involvement primarily isolated to the optic nerve in our patient cohort.

This study has some inherent limitations due to its retrospective design. One potential limitation is the risk of recall bias, which may result in inaccurate grouping of participants, particularly with regards to the time relationship between COVID-19 and ON onset, although detailed information on prior COVID-19 infection was recorded at the time of admission. Additionally, the relatively small sample size may limit the generalizability of the findings. Furthermore, to gain a deeper understanding, it’s vital to include other infection-control groups in comparisons, such as examining the incidence rates of MOG-ON and AQP4-ON during influenza seasons. This would offer insight into whether COVID-19 associated ON presents distinct traits or is merely a usual manifestation of post-infection MOG-ON, considering that MOG-ON/MOGAD might be linked to non-specific viral infections. A follow-up study is also needed to provide information on the long-term outcomes of the patients.

## 5 Conclusion

Mild COVID-19 is more likely associated with MOG-ON than AQP4-ON. MOG-ON that develops within 6 weeks following a COVID-19 infection may be associated with the COVID-19 infection. Comorbidities such as hypertension, diabetes, and AVDs may have a synergistic effect on MOG-ON in patients with COVID-19, which warrants further investigation. COVID-19 related MOG-ON often affects both eyes, and acute visual function damage can be severe, but generally has a good prognosis.

## Data availability statement

The original contributions presented in the study are included in the article/supplementary material, further inquiries can be directed to the corresponding author.

## Ethics statement

The studies involving humans were approved by Beijing Tongren Hosipital, Capital Medical University. The studies were conducted in accordance with the local legislation and institutional requirements. The participants provided their written informed consent to participate in this study. Written informed consent was obtained from the individual(s) for the publication of any potentially identifiable images or data included in this article.

## Author contributions

LS: Writing – review & editing, Writing – original draft, Validation, Software, Project administration, Methodology, Investigation, Data curation, Conceptualization. JW: Formal analysis, Conceptualization, Writing – review & editing, Methodology. QY: Formal analysis, Writing – review & editing. YG: Writing – original draft, Conceptualization, Formal analysis, Writing – review & editing, Supervision.
